# JNK signaling provides a novel therapeutic target for Rett syndrome

**DOI:** 10.1186/s12915-021-01190-2

**Published:** 2021-12-16

**Authors:** Clara Alice Musi, Anna Maria Castaldo, Anna Elisa Valsecchi, Sara Cimini, Noemi Morello, Riccardo Pizzo, Alessandra Renieri, Ilaria Meloni, Maurizio Bonati, Maurizio Giustetto, Tiziana Borsello

**Affiliations:** 1 Department of Pharmacological and Biomolecular Sciences, Milan University, Via Balzaretti 9, 20133 Milan, Italy; 2 Department of Neuroscience, Istituto di Ricerche Farmacologiche Mario Negri-IRCCS, Via Mario Negri 2, 20156 Milan, Italy; 3 Recordati S.p.A., Drug Discovery, Via Civitali, Milano, Italy; 4 Department of Neuroscience and National Institute of Neuroscience, University of Turin, Turin, Italy; 5 Medical Genetics, University of Siena, Siena, Italy; 6 Department of Public Heath, Istituto di Ricerche Farmacologiche Mario Negri-IRCCS, Milan, Italy

**Keywords:** MECP2, Apnea, Synaptic dysfunction, D-JNKI1, Neurodevelopmental disease, Neuroprotection

## Abstract

**Background:**

Rett syndrome (RTT) is a monogenic X-linked neurodevelopmental disorder characterized by loss-of-function mutations in the MECP2 gene, which lead to structural and functional changes in synapse communication, and impairments of neural activity at the basis of cognitive deficits that progress from an early age. While the restoration of MECP2 in animal models has been shown to rescue some RTT symptoms, gene therapy intervention presents potential side effects, and with gene- and RNA-editing approaches still far from clinical application, strategies focusing on signaling pathways downstream of MeCP2 may provide alternatives for the development of more effective therapies in vivo. Here, we investigate the role of the c-Jun N-terminal kinase (JNK) stress pathway in the pathogenesis of RTT using different animal and cell models and evaluate JNK inhibition as a potential therapeutic approach.

**Results:**

We discovered that the c-Jun N-terminal kinase (JNK) stress pathway is activated in Mecp2-knockout, Mecp2-heterozygous mice, and in human MECP2-mutated iPSC neurons. The specific JNK inhibitor, D-JNKI1, promotes recovery of body weight and locomotor impairments in two mouse models of RTT and rescues their dendritic spine alterations. Mecp2-knockout presents intermittent crises of apnea/hypopnea, one of the most invalidating RTT pathological symptoms, and D-JNKI1 powerfully reduces this breathing dysfunction. Importantly, we discovered that also neurons derived from hiPSC-MECP2 mut show JNK activation, high-phosphorylated c-Jun levels, and cell death, which is not observed in the isogenic control wt allele hiPSCs. Treatment with D-JNKI1 inhibits neuronal death induced by MECP2 mutation in hiPSCs mut neurons.

**Conclusions:**

As a summary, we found altered JNK signaling in models of RTT and suggest that D-JNKI1 treatment prevents clinical symptoms, with coherent results at the cellular, molecular, and functional levels. This is the first proof of concept that JNK plays a key role in RTT and its specific inhibition offers a new and potential therapeutic tool to tackle RTT.

**Supplementary Information:**

The online version contains supplementary material available at 10.1186/s12915-021-01190-2.

## Background

Rett syndrome (RTT) is a rare disease and is one of the most common genetic causes of cognitive impairment in women [1]. De novo mutations within the gene encoding for methyl CpG-binding protein2 (MeCP2), in the X chromosome, are the genetic basis of most cases of Rett syndrome [2]. MeCP2 is a ubiquitous nuclear protein, abundantly expressed in the central nervous system (CNS), which has been identified by Bird and co-authors as a protein that binds methylated CpG dinucleotides [3, 4].

RTT is a progressive neurological disorder: patients usually achieve normal neurodevelopmental milestones, motor functions, and communication skills, but between 8 and 36 months of age regression starts with cognitive impairments that progress with many other severely disabling problems [5, 6]. These include growth failure, epilepsy, gastrointestinal disorders, scoliosis, and cardio-respiratory abnormalities [7–9]*.* The main neurological defect is the reduction in brain weight (12–34%) with changes in the structural and molecular organization of synapses [10, 11]. Recent studies report that subtle changes in connectivity and communication among neurons [12], density, stability, and turnover of dendritic spines is altered in RTT brains [13]. There is also a shift of the excitatory/inhibitory balance, observed both in murine and human models [14], that helps to explain the RTT-brain defects [15–17]. Furthermore, the brainstem presents defects in synaptic transmission [18] and this is in line with the breathing disturbances consistently observed in RTT [19, 20]. Importantly, the restoration of the gene in Mecp2-defective mice can rescue most of the symptoms, indicating that RTT can be reversed and that MeCP2 is required for normal neuronal function throughout adult life [21]. Although RTT is potentially curable, at present, there are no effective treatments. Gene therapy, which is an ideal intervention for monogenic diseases, presents many side effects and correction of the mutations by gene- or RNA-editing methods is still far from translation into the clinic. Alternative therapeutic approaches to gene therapy require the identification of intracellular pathways that are deregulated in RTT. Interfering with key proteins downstream MeCP2, by bioactive tools, could lead to the development of the effective therapeutic intervention in vivo.

In the CNS, MeCP2 loss results in the deregulation of different intracellular pathways divided into three categories: (1) neurotransmitters and neuromodulators (monoamines, glutamate, and acetylcholine), (2) growth factors (BDNF and IGF-1), and (3) metabolic and stress signaling (lipids, glucocorticoids, and oxidative stress) [22].

The c-Jun-N-terminal kinase (JNK) signaling transduction pathway is an intracellular pathway strongly responsive in the brain to different stressors and plays a key role in brain development and maintenance [23, 24]. JNK regulates neuronal migration, brain morphogenesis, and axon-dendritic architecture during development [24] and governs fundamental functions in the adult brains. Mammals express three JNK isoforms: JNK1, JNK2, and JNK3, and single-knockout mice for JNK1, JNK2, and JNK3 present severe CNS defects [24]. In the adult brain, JNK controls neuronal plasticity and memory formation [24, 25], synaptic functions [26–28], neuronal death [29, 30], and mediates neuro-inflammation [31]. The JNK signaling pathway is involved in many brain diseases such as stroke [32, 33], epilepsy [34, 35], Alzheimer’s [26, 36–38], Parkinson’s [39–41], and Huntington’s diseases [42] as well as spinal muscular atrophy [43].

Less is known about JNK’s function in neurodevelopmental diseases such as autism spectrum disorders, which, however, are characterized by synaptic alterations as well. We investigated the activation profile of the JNK pathway in three different RTT models: (1) Mecp2-knockout (Mecp2^y/−^) male mice from the Bird group; (2) Mecp2-heterozygous female mice (Mecp2^+/− Jae^) developed by Jaenisch, with exon 3 deletion in Mecp2; and (3) human neurons, differentiated from human MECP2-mutated iPSCs (MECP2^mut^) compared to the isogenic control expressing wild-type MECP2 allele (MECP2^wt^).

We decided to focus on three different brain regions correlated to the major pathological symptoms display in Rett: cortex, hippocampus, and cerebellum.

JNK was powerfully activated in male and female mutant mice and in human neurons differentiated from MECP2^mut^ iPSCs, while in MECP2^wt^ iPSCs was not. By inhibiting JNK, using the cell-permeable JNK inhibitor peptide D-JNKI1 [33, 44] (commercial name XG101/AM111), Mecp2-knockout and heterozygous RTT mouse phenotypes were rescued and the cell death induced in MECP2^mut^ hiPSCs was prevented. These results suggest that JNK inhibition could offer an attractive therapeutic strategy to tackle RTT.

## Results

### Mecp2^y/−^ Bird male mice mimic severe and acute neurological RTT signs

**Fig. 1:**
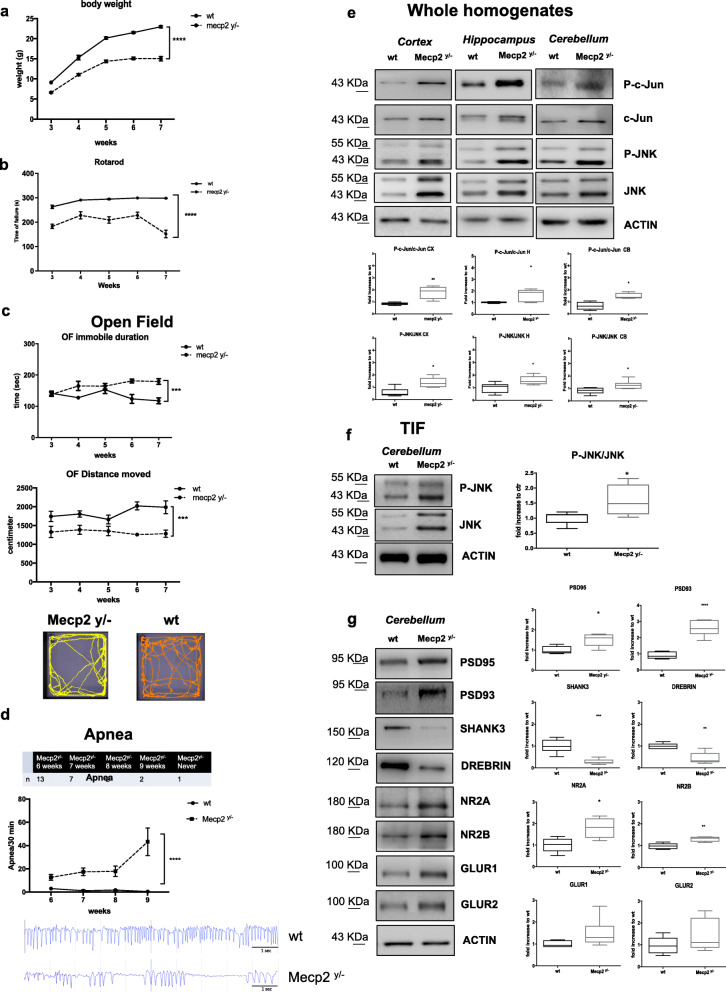
JNK signaling activation in Mecp2^y/^^-^ Bird male mice. **a** Growth curves of Mecp2^y/^^-^ mice (*n*=25) compared to their wt littermates (*n*=25) from 3 to 7 weeks of age. **b, c** Behavioral analysis of Mecp2^y/^^-^ mice (*n*=10) compared to their control wt (*n*=10) from 3 to 7 weeks of age in the Rotarod (**b**) and open field (**c**) tests (parameters shown: duration of immobility and distance moved). The distance moved by each experimental group was also presented in the arena plots under the open field graphs. **d** On-set and number of apnea in Mecp2^y/^^-^ and wt mice at 6, 7, 8, and 9 weeks of ages in the table and the graphs, in the lower part, the representative plethysmographic traces of Mecp2^y/−^ (*n*=15) vs wt (*n*=6) characterizing the respiratory patterns and breathing dysfunction. **e** JNK signaling pathway activation in the whole homogenate: Western blots and quantifications of P-c-Jun/c-Jun and P-JNK/JNK ratios in the cortex, hippocampus, and cerebellum of 7-week-old Mecp2^y/^^-^ (*n*=10) and wt (*n*=10) mice. **f** Western blots and quantifications of TIF fraction (post-synaptic elements) showed the JNK activation in cerebellum of 7-week-old Mecp2^y/^^-^ (*n*=10) compared to wt (*n*=10) mice. **g** Western blots and quantifications showed PSD alterations in Mecp2^y/^^-^ (*n*=10) compared to wt (*n*=10) mice. Data were shown as mean ± SEM. Significance was calculated using two‐way ANOVA for repetitive measurements followed by Bonferroni post hoc test (panels **a**, **b**, **c**, and **d**) or Student’s *t* test followed by Tukey’s post hoc test (panels **e**, **f**, **g**). Statistical significance: *p<0.05, **p<0.01, ***p<0.001, and ****p<0.0001

The Mecp2 Bird (Mecp2tm1.1Bird) [45] mouse model presented a reduction of fertility associated with Mecp2 mutation and with poor breeding performance. Pups had high mortality (15%) and a low rate of transgenic animal births (15% of heterozygous Mecp2^+/−^, 24% of homozygous Mecp2^y/−^, and 46% wild-type mice). Male Mecp2^y/−^ mice had a fast, severe, and acute onset of RTT, starting at 3/4 weeks of age, leading very early to death (at 7/10 weeks of age), while Bird females died later at 36 weeks of age [45, 46]. We used male Mecp2^y/−^ to mimic a severe RTT model [47]. Mecp2^y/−^ mice were monitored from 3 to 7 weeks of age, analyzing their well-being conditions, focusing on the bodyweight growth curves and food and water intake. Bodyweight (*p*<0.0001, Fig. 1a) dropped deeply in Mecp2^y/−^ compared to wild-type (wt) mice, related to worsening of the neurological symptoms (see Tables 1 and 2).

**Table 1:** Food intake in wt and Mecp2^y/−^ male mice. Food intake did not change between wt and Mecp2^y/−^ mice

Water intake	Wt (g)		Mecp2^**y/**−^ (g)		Significance
Weeks	Mean	SEM	Mean	SEM	Wt vs Mecp2^y/−^
3	4.6283	0.4713	2.7983	0.4139	*
4	5.3220	0.5939	3.0389	0.2847	**
5	5.9850	0.5199	3.0300	0.3300	****
6	5.1850	0.6216	2.6943	0.2691	***
7	3.8911	0.4108	3.2786	0.3967	n.s.

Data were shown as mean and SEM. Significance was calculated using two‐way ANOVA followed by Bonferroni post hoc test. Significance relative to control *p<0.05, **p<0.01, ***p<0.001, and ****p<0.0001

**Table 2:** Water intake in wt and Mecp2^y/−^ male mice. Water intake is strongly reduced in Mecp2^y/−^ mice compared to wt at 3, 4, 5, and 6 weeks of age but recover in the last time point analyzed

Food intake	Wt (g)		Mecp2^**y/**−^ (g)		Significance
Weeks	Mean	SEM	Mean	SEM	Wt vs Mecp2^y/−^
3	3.4033	0.2398	2.7093	0.4323	n.s.
4	3.6329	0.2513	3.1867	0.2859	n.s.
5	4.0980	0.1611	3.6411	0.2162	n.s.
6	3.2463	0.2695	3.3770	0.1990	n.s.
7	3.4764	0.2690	3.3400	0.3826	n.s.

Data were shown as mean and SEM. Significance was calculated using two‐way ANOVA followed by Bonferroni post hoc test. Significance relative to control *p<0.05, **p<0.01, ***p<0.001, and ****p<0.0001

We did not observe any significant change in food intake between wt and Mecp2^y/−^ mice (Table 1). On the contrary, Mecp2^y/−^ mice consume less water compared to age-matched wt in the first time points analyzed (*p*<0.05, *p*<0.01, *p*<0.001, *p*<0.0001, Table 2). These differences disappear at 7 weeks of age, a time point in which the water consumption is comparable between the two groups (Table 2).

Locomotion in Mecp2^y/−^ mice was investigated in the Rotarod and open field tests, repeated each week from 3 to 7 weeks of age. On the Rotarod Mecp2^y/−^, mice had a shorten latency on the wheel than wt animals, with a significant decrease of locomotor ability at each time point (*p*<0.0001, Fig. 1b). Locomotor performance was further investigated in the open field test by analyzing the distance moved and the time spent immobile in the arena. Mecp2^y/−^ mice spent significantly more time immobile (*p*=0.0001, Fig. 1c) and consequently less distance moved (*p*=0.0003, Fig. 1c) than wt mice. These data confirm the severe and well-detectable locomotor impairments in Mecp2^y/−^ mice compared to age-matched wt mice. Breathing dysfunction in Mecp2^y/−^ mice was tested by whole-body plethysmography to quantify frequency (f), time of the inspiration (T_i_), and expiration (T_e_), and apnea over 30 min of freely moving recording from 6- to 9-week-old Mecp2^y/−^ mice. The onset and progress of respiratory dysfunction varied among Mecp2^y/−^ mice examined. In the majority (13 out of 25) of mice, apnea appeared at 6 weeks of age, but 7 showed apnea at 7 weeks, 2 at 8 weeks, 2 at 9 weeks, and 1 died without a single episode (Fig. 1d, table). Our results show that apnea started appearing at 6 weeks and became more frequent with age (*p*<0.0001; Fig. 1d).

### Mecp2^y/−^ Bird mice present activation of the JNK pathway and dendritic spine alterations

To analyze the activation of the JNK stress pathway in Mecp2^y/−^ Bird mice, we investigated the phosphorylation of c-Jun (P-c-Jun levels over c-Jun total levels: P-c-Jun/c-Jun) and JNK (P-JNK levels over JNK total levels: P-JNK/JNK) in three brain areas: cortex, hippocampus, and cerebellum. P-c-Jun/c-Jun and P-JNK/JNK ratios were significantly higher in Mecp2^y/−^ than age-matched wt mice (Fig. 1e), indicating powerful activation of the JNK-stress pathway. In the Mecp2^y/−^ mice, the P-c-Jun/c-Jun ratio was 50% higher in the cortex (*p*=0.0055, Fig. 1e), 75% in the hippocampus (*p*=0.02, Fig. 1e), and 50% in the cerebellum (*p*=0.03, Fig. 1e) than in wild-type mice. Thus, in these brain regions, the phosphorylation of JNK was significantly higher in Mecp2^y/−^ than age-matched wt mice (cortex: *p*=0.013, hippocampus: *p*=0.03, cerebellum: *p*=0.028, Fig. 1e).

In addition, to further demonstrate the JNK signaling implication in motor and breathing dysfunction, we performed p-c-jun immunostaining on the brainstem of wild-type and Mecp2^y/−^ mice at 7 weeks of age. This area presented some regions with strong immunolabeling for p-c-jun in Mecp2^y/−^ compared to wt mice (Additional File 1: Fig. S1), suggesting the involvement of the JNK pathway in these impaired functions.

The JNK pathway is associated with neuronal death, synaptic dysfunction [24, 26–28], and dendritic spine dysgenesis [16]. We specifically assessed JNK activation at dendritic spines, by isolating the postsynaptic-enriched protein fraction with the triton-insoluble fraction (TIF) in the cerebellum because it regulates thin movements, motor-spatial memory, and motor coordination and is thus relevant to RTT locomotor impairments.

The P-JNK/JNK ratio was significantly higher in Mecp2^y/−^ than wt mice (*p*=0.027, Fig. 1f), indicating JNK activation in the post-synaptic protein enriched-fraction. To study the molecular organization of dendritic spines in Mecp2^y/−^ mice, we quantified the levels of different postsynaptic markers. PSD95 (25%) and PSD93 (70%) levels were higher in Mecp2^y/−^ mice (*p*=0.0149 and *p*<0.0001, Fig. 1g), while SHANK3 (70%) and Drebrin (60%) levels were lower than in wt mice (*p*=0.001, *p*=0.0016, Fig. 1g). In addition, *N*-methyl-d-aspartate receptor 2A (GluN2A) and 2B (GluN2B) levels were significantly higher in Mecp2^y/−^ than in wt mice (60%, *p*=0.03 and 37%, *p*=0.01, Fig. 1g). Finally, the levels of GluA1 and GluA2, AMPA receptor subunits, presented a tendency not significant to be higher in Mecp2^y/−^ than in wt mice (Fig. 1g). These results indicate abnormal expression and/or organization of the PSD region in the absence of MeCP2.

### The specific JNK inhibitor peptide D-JNKI1 rescues the severe and acute neurological RTT signs in Mecp2^y/−^ Bird male mice

#### D-JNKI1 efficacy inhibits the JNK pathway in Mecp2^y/−^ mice

**Fig. 2:**
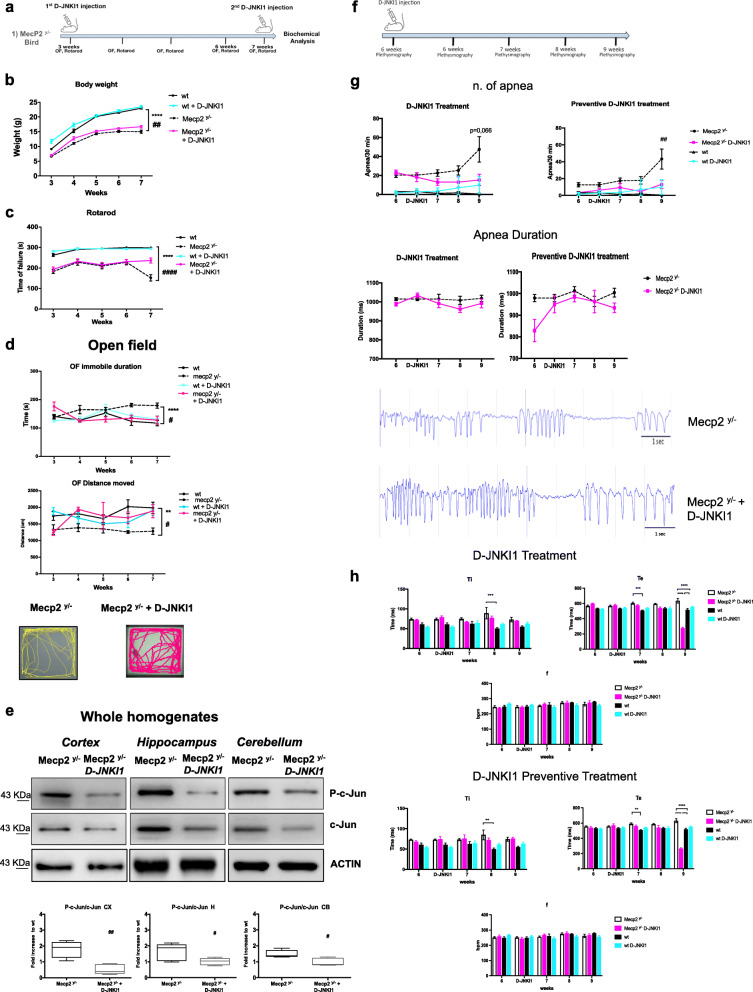
D-JNKI1 rescues well-being conditions, locomotor impairments, and apnea numbers in Mecp2^y/^^-^ male mice. **a** Timeline of D-JNKI1 treatment in *Mecp2*^*y/*^^-^ *male mice*. **b** Growth curves of D-JNKI1-treated (blue sky) and untreated (black) wild type, and D-JNKI1-treated (fuchsia) and untreated (black-dotted) Mecp2^y/-^ mice from 3 to 7 weeks of age (*n*=25 for each experimental group). **c, d** Behavioral analysis of D-JNKI1-treated vs untreated wt and Mecp2^y/^^-^ mice (*n*=10 for each experimental group) from 3 to 7 weeks of age in the Rotarod (**c**) and open field (**d**) tests (parameters shown: time spent immobile and distance moved, with relative open field arena-plots). **e** Western blots and the quantification P-c-Jun/c-Jun ratio in the whole homogenate of the cortex, hippocampus, and cerebellum of 7-week-old D-JNKI1-treated and untreated Mecp2^y/^^-^ mice. **f** Timeline of D-JNKI1 treatment and plethysmography analysis. **g** Number and duration of apnea in preventive and curative D-JNKI1 paradigm of treated (*n*=10) and untreated (*n*=6) wild type and Mecp2^y/^^-^ mice (*n*=15) from 6 to 9 weeks of age. **h** Breathing analysis in preventive (lower part) and curative (upper part) D-JNKI1 paradigm of treated (fuchsia) and untreated Mecp2^y/^^-^ (white) mice from 6 to 9 weeks of age and the treated (blue-sky) and untreated wild type (black) (parameters shown: Ti, Te, and f). Data were shown as mean ± SEM. Significance was calculated using two‐way ANOVA for repetitive measurements followed by Bonferroni post hoc test (panels **a**, **b**, **c**, **e**, **f**) or Student’s *t* test followed by Tukey’s post hoc test (panels **d**). Statistical significance relative to control **p<0.01, ***p<0.001, and ****p<0.0001; D-JNKI1-treated vs untreated Mecp2^y/^^-^: #*p*<0.05, ##*p*<0.01, ####*p*<0.0001

The effectiveness and specificity of the cell-permeable peptide D-JNKI1 treatment were measured by its inhibitory effect on c-Jun in vivo [33]. The P-c-Jun/c-Jun ratio was therefore measured in the cortex, hippocampus, and cerebellum. D-JNKI1 treatment powerfully prevented c-Jun phosphorylation in the cortex (84%), hippocampus (50%), and cerebellum (36%, Fig. 2e) in treated compared to untreated Mecp2^y/−^ mice (*p*=0.0011, *p*=0.0482, *p*=0.0204, Fig. 2e). These results confirm the inhibitory specificity of D-JNKI1’s action.

#### D-JNKI1 improves the well-being conditions and rescues locomotor impairments

The D-JNKI1 peptide [33, 44] was injected intraperitoneally from 3 to 7 weeks of age, every 28 days to prevent JNK hyper-activation in Mecp2^y/−^ mice (Fig. 2a). This protocol of treatment was set up based on previous publications—see “Methods” section. By comparing the well-being conditions between treated and untreated Mecp2^y/−^ mice, D-JNKI1 chronic treatment did not cause any major toxic or side effects nor body weight loss and changes in food and water assumptions (Tables 3 and 4), on the contrary, induced a recovery in body weight (wt vs Mecp2^y/−^ *p*<0.0001; D-JNKI1-treated vs untreated Mecp2^y/−^ *p*=0.0020, Fig. 2b).

**Table 3:** Food consumption in treated and untreated wt and Mecp2^y/−^ male mice. The D-JNKI1 treatment did not affect the food assumption in wt as well as in Mecp2^y/−-^treated mice

Food intake	Wt (g)		Wt + D-JNKI1 (g)		Mecp2^**y/**−^ (g)		Mecp2^**y/**−^ + D-JNKI1(g)		Significance	
weeks	Mean	SEM	Mean	SEM	Mean	SEM	Mean	SEM	Wt vs wt+D-JNKI1	Mecp2^y/−^ vs Mecp2^y/−^ +D-JNKI1
3	3.4033	0.2398	3.4920	0.3657	2.7093	0.4323	3.5675	0,4089	n.s.	n.s.
4	3.6329	0.2513	3.6067	0.2246	3.1867	0.2859	3.4183	0,2000	n.s.	n.s.
5	4.0980	0.1611	3.6975	0.0727	3.6411	0.2162	3.9000	0,5425	n.s.	n.s.
6	3.2463	0.2695	3.8471	0.2211	3.3770	0.1990	2.4700	0,1815	n.s.	n.s.
7	3.4764	0.2690	4.4220	0.3107	3.3400	0.3826	3.3300	0,3169	n.s.	n.s.

Data were shown as mean and SEM

**Table 4:** Water consumption in treated and untreated wt and Mecp2^y/−^ male mice. D-JNKI1 did not change the water intake in both wt and Mecp2 ^y/−^-treated mice

Water intake	Wt (g)		Wt + D-JNKI1 (g)		Mecp2^**y/**−^ (g)		Mecp2^**y/**−^ + D-JNKI1(g)		Significance	
Weeks	Mean	SEM	Mean	SEM	Mean	SEM	Mean	SEM	Wt vs wt+D-JNKI1	Mecp2^y/−^ vs Mecp2^y/−^ +D-JNKI1
3	4.6283	0.4713	5.5329	0.9571	2.7983	0.4139	4.5100	1,4200	n.s.	n.s.
4	5.3220	0.5939	5.6950	0.6502	3.0389	0.2847	2.8580	0,2901	n.s.	n.s.
5	5.9850	0.5199	5.3333	0.3999	3.0300	0.3300	2.6360	0,2393	n.s.	n.s.
6	5.1850	0.6216	4.5763	0.3882	2.6943	0.2691	3.2700	0,4998	n.s.	n.s.
7	3.8911	0.4108	4.3420	0.5317	3.2786	0.3967	2.8500	0,6756	n.s.	n.s.

Data were shown as mean and SEM

Additionally, D-JNKI1-treated Mecp2^y/−^ mice had a significant lower latency to fall in the Rotarod test (*p*<0.0001, Fig. 2c) and also better open field test performances (Fig. 2d). The time spent in an immobile state was shorter and the distance moved greater (*p*=0.018 and *p*=0.0237, Fig. 2d) in D-JNKI1-treated compared to untreated Mecp2^y/−^ mice. D-JNKI1 did not show any toxic effects in wild-type mice, and the curves of D-JNKI1-treated and untreated wt animals overlapped with no significant differences (Fig. 2b–d).

#### D-JNKI1 reduces apnea numbers

Due to the onset variability of apnea among Mecp2^y/−^, we used two different paradigms of D-JNKI1 treatment: preventive and curative administration (Fig. 2f). In the first group, Mecp2^y/−^ were treated at 6 weeks of age, before the onset of apnea, to analyze the potential preventive effect of D-JNKI1, whereas in the second group, mice received the treatment only after showing the first apnea, a protocol set to provide information on the potential curative effect of D-JNKI1. All mice were recorded at 6 weeks of age and again the day after the D-JNKI1 treatment.

Using whole-body plethysmography, we monitored breathing patterns (frequency (f), time of inspiration (T_i_) and expiration (T_e_), and apnea (end-expiratory pause greater than 800 ms)) in five different experimental mice groups: untreated Mecp2^y/−^, Mecp2^y/−^ preventive-treated, Mecp2^y/−^ curative-treated, and treated and untreated wt mice from 6 up to 9 weeks of age. The preventive D-JNKI1 treatment induces a significant decrease in apnea numbers (*p*=0.0047, Fig. 2g) but not in their duration, while the curative treatment exerts an almost significant effect in the reduction of apnea numbers but not in their duration in Mecp2^y/−^ mice (*p*=0.066; Fig. 2g). We observed no significant differences among all groups in breathing rate (f). On the other hand, T_e_ presented differences between wt and Mecp2^y/−^ at 7 weeks in both groups of curative and preventive D-JNKI1 treatment (*p*=0.0002, *p*=0.0035, Fig. 2h); this is in line with the fact that these mice already presented apnea. Genotypic differences of T_i_ were clear at 8 weeks in both groups (*p*=0.0002 and *p*=0.0025, Fig. 2h) but both curative and preventive treatments were ineffective. Finally, at 9 weeks, the last time point tested, the genotypic effect was clear and both the curative and preventive D-JNKI1 treatments significantly reduced the T_e_ (*p*<0.0001; Fig. 2h). For T_i_ neither curative nor preventive D-JNKI1 treatments had any effect. Concerning the D-JNKI1 treatment in wt mice, this did not show any significant effect.

#### D-JNKI1 effect on dendritic spine alterations in Mecp2^y/−^

**Fig. 3:**
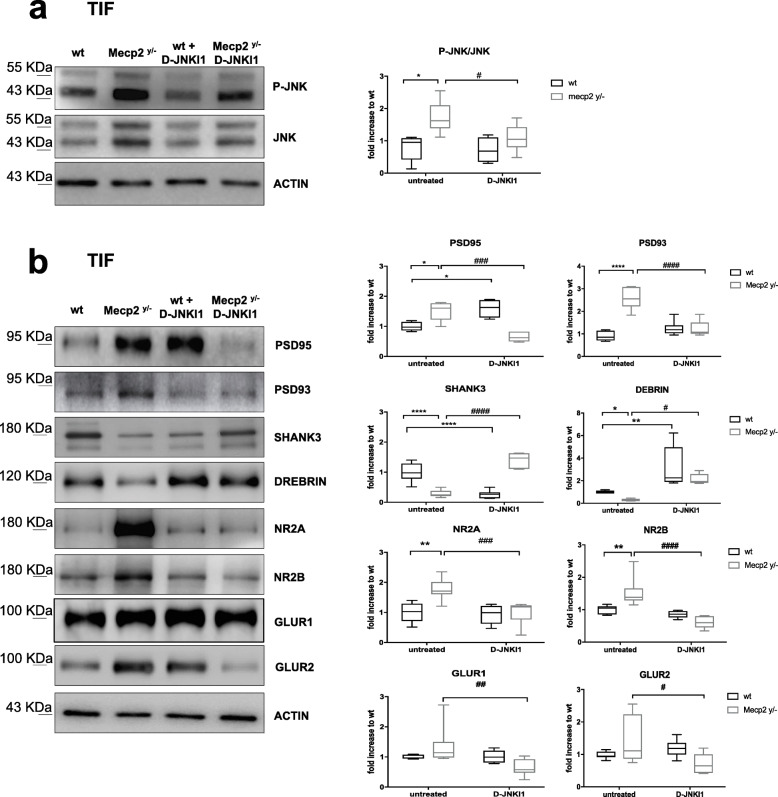
D-JNKI1 effect against dendritic spine alterations in Mecp2^y/-^ male mice. **a** Western blots and quantifications in the cerebellum TIF (post-synaptic elements) of treated and untreated wt and Mecp2^y/-^ mice to measure D-JNKI1 effect in vivo. D-JNKI1 significantly reduced JNK activation in Mecp2^y/^^-^ mice, but not in control wt mice. **b** Western blots and quantifications of the post-synaptic elements in the cerebellum showed normalization of the PSD markers levels to control level in D-JNKI1-treated compared to untreated Mecp2^y/^^-^ mice (*n*=10 for each experimental group). Data were shown as mean ± SEM. Significance was calculated using two‐way ANOVA followed by Bonferroni post hoc test. Significance relative to control *p<0.05, **p<0.01, and ****p<0.0001. D-JNKI1-treated vs untreated Mecp2^y/^− #*p*< 0.05, ##*p*< 0.01, ###*p*<0.001, and ####*p*<0.0001

D-JNKI1 inhibitor also rescued the changes in postsynaptic markers observed in Mecp2^y/−^ mice. The treatment strongly lowered the P-JNK/JNK ratio in the postsynaptic enriched protein fraction (TIF) (equal to 40%) in Mecp2^y/−^ (*p*=0.047, Fig. 3a) compared to untreated mice. The Mecp2^y/−^ mice had high PSD95 and PSD93 levels (*p*=0.018 and *p*<0.0001 Fig. 3b), while SHANK3 and Drebrin decreased (*p*<0.0001 and *p*=0.049, Fig. 3b), D-JNKI1 restored PSD95 (*p*=0.0002, Fig. 3b), and PSD93 (*p*<0.0001, Fig. 3b) level to 75% and 65% and SHANK3 and Drebrin level to 80 and 90% (*p*<0.0001 and *p*=0.0121, Fig. 3b), normalizing the biochemical marker levels of Mecp2^y/−^.

The inhibitor treatment-induced recovery of NMDA and AMPA receptors to wt levels. GluN2A decreased 40% (*p*=0.0008, Fig. 3b), GluN2B 58% (*p*<0.0001, Fig. 3b), GluA1 50% (*p*=0.0082, Fig. 3b), and GluA2 50% (*p*=0.0113, Fig. 3b) compared to untreated Mecp2^y/−^ mice. In wild-type-treated mice, D-JNKI1 significantly increased PSD95 and Drebrin levels (*p*=0.0107, *p*=0.00021 Fig. 3b) and lowered SHANK3 (*p*<0.0001, Fig. 3b), however without any effect on glutamate receptors (AMPA and NMDA). The effects of D-JNKI1 on PSD markers in wt mice did not modify their behavioral performances, thus excluding major toxic effects.

### The specific JNK inhibitor peptide D-JNKI1 rescues the milder neurological RTT signs in Mecp2^+/− Jae^ heterozygous mice

Mecp2^+/− Jae^ (Mecp2tm1.1Jae) heterozygous female mice, carrying a deletion on exon 3 in Mecp2, mimic a milder RETT phenotype that well recapitulates the somatic mosaicism of Mecp2 mutation reported in RTT patients and develop later RTT patient symptoms including irregular breathing, abnormal gait, and hind limb clasping [48].

**Fig. 4:**
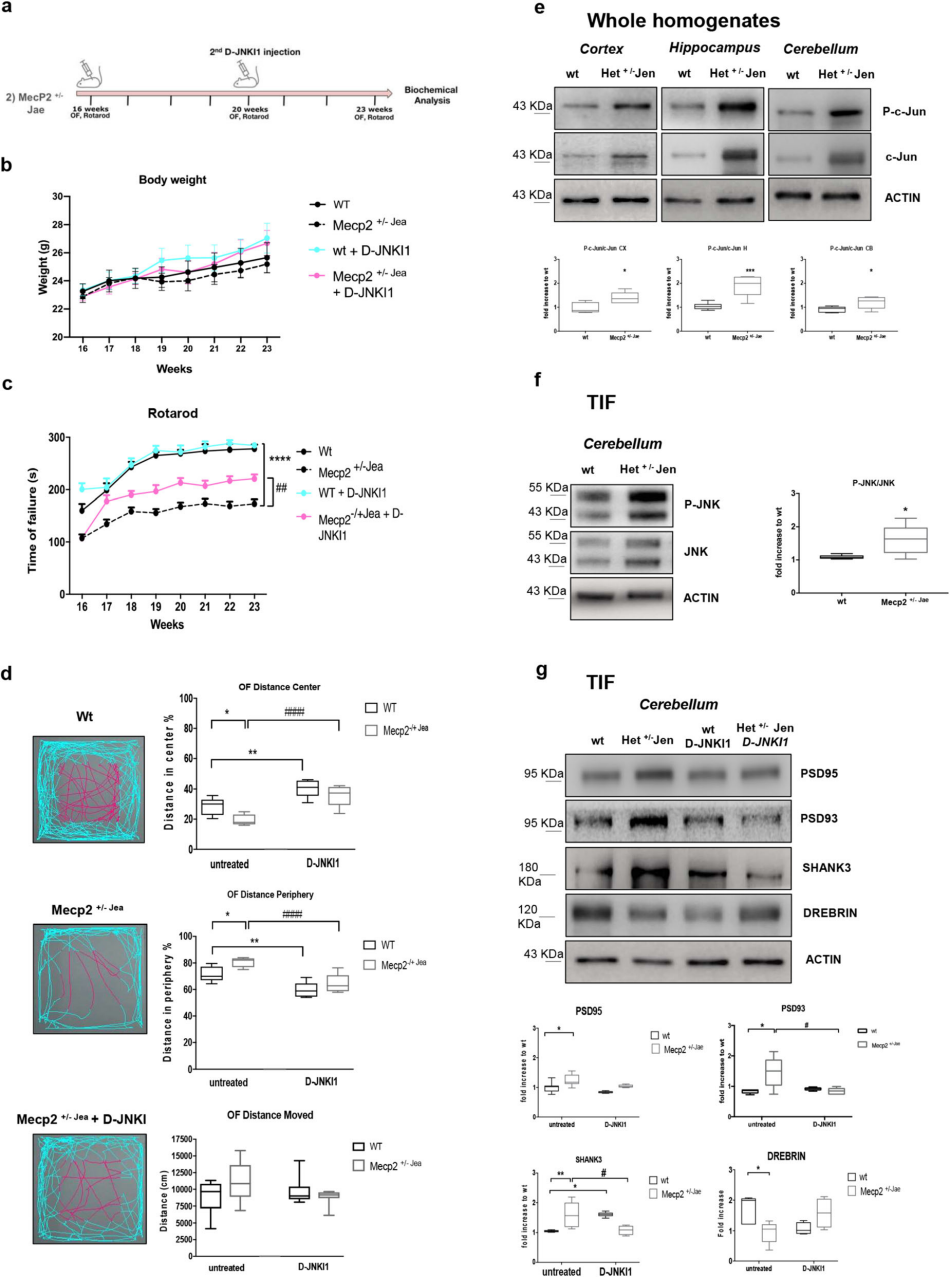
Female Mecp2^+/−^ Jaenisch neurological phenotype: JNK signaling activation and D-JNKI1 treatment. **a** Timeline of D-JNKI1 treatment in *Female Mecp2*^+/−^ *Jaenisch mice.* **b** Growth curves of D-JNKI1-treated and untreated wt and Mecp2^+/− Jae^ mice from 16 to 23 weeks of age. **c** Rotarod tests in D-JNKI1 Mecp2^+/− Jae^-treated mice (fuchsia line), Mecp2^+/− Jae^ untreated (black dotted line), treated wt (blue-sky line), and untreated wt (black line). **d** Open field test. D-JNKI1 improved the behavioral performance of Mecp2^+/− Jae^ (see plots for central (fuchsia) and peripheral (blue sky) movements of wt and Mecp2^+/− Jae^-treated and untreated mice). The last graph presented the distance moved: there were no genotypic differences. **e** Western blots and quantifications of c-Jun activation in the whole homogenate of the cortex, hippocampus, and cerebellum in 23-week-old wt and Mecp2^+/− Jae^ mice. **f** Western blots and relative quantifications in the TIF cerebellum of 23-week-old wt and Mecp2^+/− Jae^ mice confirmed JNK activation at the synaptic level in Mecp2^+/− Jae^ mice. **g** Mecp2^+/− Jae^ presented alterations of the PSD-region and D-JNKI1 treatment normalized the biochemical alterations in treated vs untreated Mecp2^+/− Jae^ mice. Each experimental group: *n*=8. Data were shown as mean ± SEM. Significance was calculated using two‐way ANOVA for repetitive measurements followed by Bonferroni post hoc test (panels **a**, **b**, **c**), Student’s *t* test followed by Tukey’s post hoc test (panels **d** and **e**), and two‐way ANOVA followed by Bonferroni post hoc test (**f**). Significant differences from control *p<0.05, **p<0.01, ***p<0.001, and ****p<0.0001; D-JNKI1-treated vs untreated Mecp2^+/−^: #*p*<0.05, ##*p*<0.01, and #### *p*<0.0001

To assess the potential protective effect of JNK inhibition in female Mecp2^+/− Jae^ mice, we treated them with D-JNKI1 depending on the developmental onset of their neurological symptoms. D-JNKI1 was delivered from 16 to 23 weeks of age, one injection every 28 days, and the behavioral tests were run every week for Rotarod test and at the end of the treatment for the open field test (Fig. 4a). Treatment did not cause any toxic side effects on the wt and Mecp2^+/− Jae^ mice metabolism, as indicated by the absence of body weight changes in each group of animals (Fig. 4b). Importantly, we found that D-JNKI1 rescued locomotor impairments in Mecp2^+/− Jae^ mice. In line with our previous observations [49], when we assessed motor coordination of female Mecp2^+/− Jae^ mice with the Rotarod test, we found that these animals showed a significant impairment compared with wt controls demonstrated by a significant shorter latency to fall off the rod (*p*<0.0001; Fig. 4c).

Intriguingly, after treatment, mutant females had a significantly longer latency to fall in the Rotarod test compared to untreated Mecp2^+/− Jae^ mice (*p*=0.0046; Fig. 4c), while no effect was observed in wt mice. In addition, in the open field test, the total distance moved did not differ between genotypes at baseline (Fig. 4d), but Mecp2^+/− Jae^ females moved less in the center of the arena (*p*=0.0134, Fig. 4d) and more in its periphery (*p*=0.022, Fig. 4d) than wt mice. Intriguingly, D-JNKI1 treatment suppressed the differences in exploratory behavior between mutant heterozygous mice and wt animals in the open field arena, as shown by the greater distance moved in the central area and a shorter distance in the peripheral area by D-JNKI1 treated than untreated Mecp2^+/− Jae^ mice (*p*<0.0001, Fig. 4d). In addition, in wild-type mice, the treatment significantly increased the distance traveled in the center of the arena and reduced the distance traveled in the periphery (*p*=0.0021 and *p*=0.0022, Fig. 4d). Thus, our data indicate that D-JNKI1 treatment improves both locomotor and exploratory defects in female Mecp2^+/− Jae^ mice.

### Mecp2^+/− Jae^ heterozygous mice present activation of the JNK pathway and dendritic spine alterations

Mecp2^+/− Jae^ females showed JNK activation (Fig. 4e), and their P-c-Jun/c-Jun ratio was 30% higher in both cortex and cerebellum (*p*=0.0141 and *p*=0.034 Fig. 4e), and 50% in the hippocampus (*p*=0.0015, Fig. 4e), compared to wt mice. The P-JNK/JNK ratio was significantly higher in dendritic spines of Mecp2^+/− Jae^ females than wt mice (*p*=0.0345, Fig. 4f), indicating that Mecp2^+/− Jae^ female mutants showed synaptic dysfunctions, as Mecp2^y/−^ male mice. The spine pathology in heterozygous females, a previously unreported defect, involved increases in PSD95 (25%), PSD93 (50%), and SHANK3 (30%) levels (*p*=0.0296, *p*=0.0308, *p*=0.0027 Fig. 4g), with a decrease in Drebrin (46%, *p*=0.0417, Fig. 4g) in the cerebellar postsynaptic enriched protein fraction. Importantly, D-JNKI1 significantly lowered both PSD93 and SHANK3 to control levels (*p*=0.0174 and *p*=0.0134, Fig. 4g), while PSD95 and Drebrin only partially returned to a normal level, with no significant effect. Finally, in wt mice, D-JNKI1 did not have any major effects on PSD proteins except for an increase of SHANK3 level (*p*=0.012 Fig. 4g), without any effect on NMDA and AMPA receptors. In sum, our biochemical results show that D-JNKI1-treatment largely rescues the molecular organization of the PSD region in Mecp2^+/− Jae^ mice.

### From animal to human iPSC models: JNK pathway activation and D-JNKI1 protective effects in Rett MECP2^mut^

**Fig. 5:**
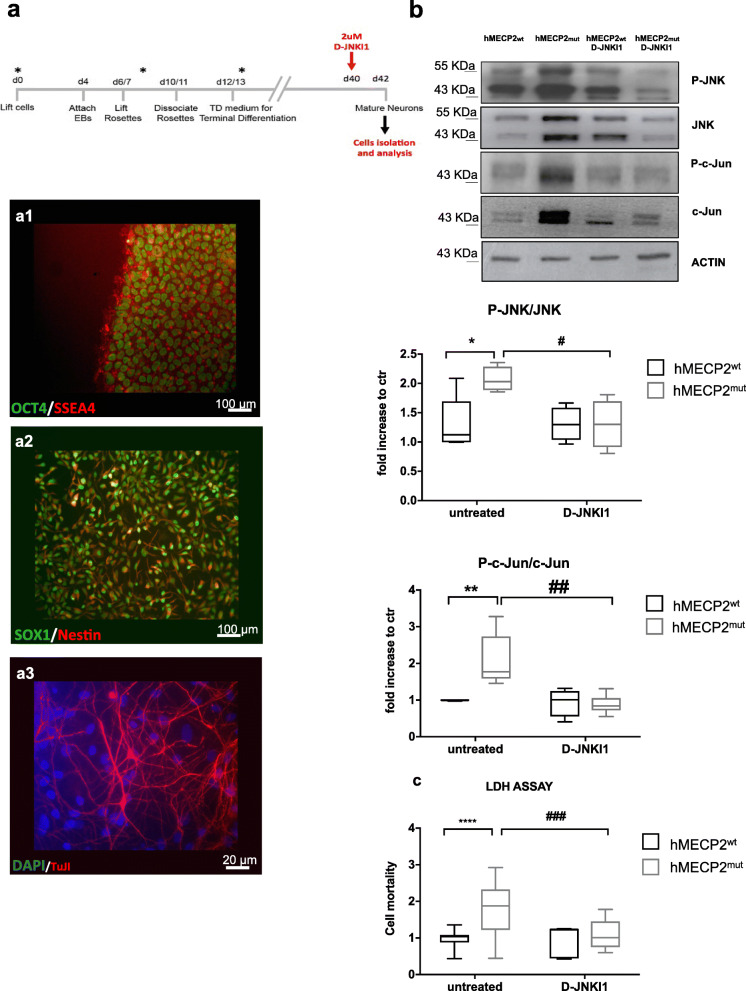
JNK signaling activation and D-JNKI1’s protective effects in Rett human iPSCs. **a** Neuronal differentiation of human iPSCs. The upper panel showed the neuronal differentiation protocol. The timing of critical steps was indicated in days from day 0 (d0). At the end of the differentiation (red arrow, d40), neurons were exposed to 2μM D-JNKI1 for 48 h before neurons were isolated for Western blot analysis. Immunofluorescence was used to define cell identity (lower panel): **a1**- OCT4/SSEA4 staining for iPSCs, **a2**- Nestin and SOX1 staining for telencephalic neural progenitors (d12/13), and **a3**- β3-Tubulin (TuJI) staining for neurons in terminally differentiated cultures and DAPI to stain nuclei. Scale bar: iPSCs and NPCs 100 μm and neurons 20 μm. **b** Western blots and quantifications of JNK activation in neurons differentiated from hiPSCs from RTT human patients; clones expressing either the wild type (hMecp2^wt^) or the mutated (hMecp2^mut^) MECP2 allele were differentiated in neurons. The hMecp2^mut^ displayed higher P-JNK/JNK and P-c-Jun/c-Jun ratios than to hMecp2^wt^ neurons. D-JNKI1 reduced hMecp2^mut^ activation to hMecp2^wt^ levels; D-JNKI1 in hMecp2^wt^ neurons did not change P-JNK/JNK (*n*=4 and 5) and P-c-Jun/c-Jun ratios (*n*=5). **c** Cell death in hMecp2^wt^ and hMecp2^mut^ neurons: the hMecp2^mut^ showed greater cell death than to hMecp2^wt^. D-JNKI1 reduced induced-cell death in the hMecp2^mut^ neurons to the control level (hMecp2^wt^). Data were shown as mean ± SEM. Significance was calculated using two‐way ANOVA followed by Bonferroni post hoc test (panel **b**). Significance vs control *p<0.05, **p<0.01, and ****p<0.0001; D-JNKI1-treated vs untreated mutated neurons #*p*<0.05, ##*p*<0.01, and ###*p*<0.001. See additional file S3

We next analyzed JNK activation in human neurons differentiated from iPSCs (hiPSCs) derived from fibroblasts of RTT patients carrying a MECP2 mutation (MECP2^mut^) and from a normal control (MECP2^wt^). We analyzed three iPSCs clones derived from fibroblasts of a female patient with Thr158Met mutation in MECP2 gene: two clones expressing the mutated MECP2 allele (2271#22 and 2271#1) and one expressing the normal allele (2271#2) due to X-chromosome inactivation, which was used as a partial isogenic control [14]. In addition, we analyzed a second MECP2-mutated patient with a p.Arg306 Cys mutation. The hiPSCs expressing either the mutated or normal MECP2 allele (hMECP2^wt^) were differentiated in cortical neurons for 30 days as previously reported, generating mainly glutamatergic neurons [14] (Fig. 5a).

Importantly, all the mutated allele hMECP2^mut^ neurons showed a powerful increase in their P-JNK/JNK and P-c-Jun/c-Jun ratios compared to hMECP2^wt^ neurons (*p*=0.0348, *p*=0.00473, Fig. 5b), providing strong evidence that MECP2 mutations activate the JNK pathway in hiPSC-derived neurons. In addition, hMECP2^mut^ neuron media had higher level of LDH (50%) than hMECP2^wt^, the internal control allele (*p*<0.0001, Fig. 5c), demonstrating that just the mutated allele induced cell death. D-JNKI1 treatment was able to reduce the P-JNK/JNK (50%) and P-c-Jun/c-Jun (70%) ratios in hMECP2^mut^ neurons, reducing their ratios to wt neurons (*p*=0.0473 and *p*=0.0015, Fig. 5b). The JNK inhibition also prevented neuronal death in hMECP2^mut^ neurons, as indicated by the significant reduction of the LDH in the media of treated compared to untreated hMECP2^mut^ neurons (*p*=0.0001, Fig. 5c).

These data showed that the JNK signaling mediates pathological alterations in human neurons differentiated from MECP2^mut^ hiPSCs. Importantly, D-JNKI1-treated control neurons did not show any increase in LDH or overt toxic effects, suggesting the safety of this treatment (Fig. 5c).

These results offer the first proof of principle that JNK is a key stress protein in human RTT and that its modulation produces therapeutic effects for this condition.

## Discussion

MECP2 mutations can lead to a variety of neurological and psychiatric problems, the most known being RTT, through a plethora of molecular and neuronal consequences. Therefore, for RTT and other MECP2-related conditions, it is important to discover the key intracellular pathways that can be targeted with drugs of high translational value [22].

We here examine stress-JNK pathway activation in three RTT models: the first, Mecp2-knockout male mice, represents a severe RTT-phenotype; the second, Mecp2-heterozygous female mice, is a milder phenotype model of this pathology but, importantly, replicates the X-linked RTT female mosaicism, and the third is a human model (hiPSCs differentiated neurons from female MECP2-RTT patients) important to assess the translational value of animal findings. The JNK pathway is activated in all three RTT models. This indicates that the Mecp2 mutation, or its absence (Mecp2^y/−^), induces downstream activation of the JNK stress-signaling pathway in both mouse and human RTT models.

Notably, the JNK pathway cross-talks with other intracellular pathways already known and more characterized in RTT, such as PI3K/AKT/GSKb/NFkB [50–52] and BDNF [53]. Its downregulation is measurable in the brain but also in saliva samples, allowing a characterization of peripheral BDNF as a biomarker [54]. Due to the inverse correlation of these two pathways, JNK and BDNF [50–53], we will investigate the effects of D-JNKI1 on BDNF levels both in central and peripheral samples in future studies.

The specific JNK inhibition, by D-JNKI1, reverses RTT pathological phenotypes in all three models. This is the first demonstration that JNK plays a key role in RTT.

D-JNKI1 modus operandi is well characterized; this peptide avoids JNK action on its JBD-dependent targets, preventing their interaction and consequently the phosphorylation. There are different JBD-dependent targets in the cell. At the dendritic spine level, PSD95 and Shank3 scaffold proteins are two of them; in the nucleus, c-Jun, that is the JNK elective target and in the cytosol, among the others, there are APP, Tau, MT-associated protein 1B (MAP1B), SCG10, BCL-2, and others [24]. D-JNKI1 is highly specific and, importantly, membrane-permeable resulting in a very potent inhibitor of JNK, without interfering with others MAPKs [33].

In symptomatic Mecp2^y/−^ male mice, there is powerful JNK activation in the total homogenate and in the postsynaptic enriched fraction (TIF) of different brain areas. It is important to an underling that synaptic abnormalities are closely correlated to the RTT symptomatology and represent the first neurodegenerative event in many diseases. Importantly, D-JNKI1 rescues the effects of the lack of Mecp2^y/−^ male mice that present the most severe RETT phenotype, improving the general well-being conditions and also significantly rescuing the behavioral defects and decreasing the apnea numbers. This effect is of note and has an important translational value, in fact, apnea occurs in 65 to 93% of all RTT patients [55, 56]. Research in mouse models of RTT suggests that different areas in the ventrolateral medulla and pons give rise to different aspects of this breathing disorder [19], but it will be necessary to study D-JNKI1 effects in these areas to explain its action.

Unfortunately, for ethical reasons (Italian national laws are very restrictive and protect animals from suffering, thus limiting the experimental time window that researchers can use when working with male Mecp2^y/−^ mice), we could not study whether the treatment prolonged the survival of male Mecp2 ^y/−^ mice treated with the peptide since we were forced to terminate the experiment when untreated mice started showing a suffering phenotype. However, it is worth noting that chronic D-JNKI1 treatment significantly improves the overall well-being condition in Mecp2^y/−^ mice. D-JNKI1 also rescues the deregulation of markers of the PSD region of the postsynaptic element in Mecp2^y/−^ mice by normalizing PSD95, PSD93, NMDA-r and AMPA-r, SHANK3, and Drebrin levels. The key role of JNK in the regulation of AMPA receptor membrane insertion [57] and in PSD95 stability [58] as well as the D-JNKI1 neuroprotective effect has been previously described in Alzheimer’s diseases [26–28, 59–61] and also in an Angelman Syndrome mouse model [62]. Here, for the first time, we link the Mecp2 absence/truncation to the activation of the JNK stress signaling pathway.

Thus, in severe RTT phenotype, D-JNKI1’s powerful neuroprotection is achieved by functional (breathing and behavior) and biochemical effects (key players of degenerative intracellular pathways). These functional improvements are important because these are a disabling aspect of RTT syndrome that dramatically impacts the patient’s quality of life.

D-JNKI1 effect was also observed in a second animal model, Mecp2^+/− Jae^ female mice, which mimic a milder RETT phenotype, expressing both the mutated and wild-type Mecp2 alleles, and thus replicating the somatic mosaicism reported in female RTT patients. Mecp2^+/− Jae^ female mice present phosphorylation of c-Jun, indicating activation of the JNK stress pathway in the cortex, hippocampus, and cerebellum of symptomatic animals. Our data provide the first indication that the molecular organization of dendritic spines is profoundly altered in Mecp2^+/− Jae^ females. These mice had atypical scaffold protein levels: PSD95, PSD93, and SHANK3 were high, while Drebrin is low, suggesting again the disorganization of the PSD region. The dysfunctional spines and these alterations are closely correlated to powerful JNK activation in the post-synaptic elements. As previously seen in Mecp2^y/−^, in Mecp2^+/− Jae^ mice too, chronic D-JNKI1 treatment improves behavioral impairments and partially rescues dendritic spine alterations, compared to untreated Mecp2^+/− Jae^ mutants. The Mecp2^+/− Jae^ display different changes in biochemical marker levels in the PSD region compared to Mecp2^y/−^; however, these are both JNK-dependent. In fact, in both models, JNK-specific inhibition restored their levels to wt animals. We speculate that this is due to the fact that JNK inhibition prevents its action on the PSD95 scaffold phosphorylation, avoiding receptor deregulation. The D-JNKI1 effect in wt mice induced a significant increase of PSD95 and a reduction in SHANK3 levels in the PSD region of the post-synaptic element, however without a correlation with the receptor levels, both NMDA and AMPA, supporting the normal behavior of control mice. In addition, preliminary experiments on hippocampal neurons in vitro treated with D-JNKI1 (2μm) for 12 days showed that the peptide-induced an increase in PSD95 level but did not significantly raise the spine number or their dimension (Borsello unpublished). Nevertheless, further investigations are needed to determine this effect in control mice.

The differences found in the molecular dis-organization of excitatory synapses in Mecp2^y/−^ and Mecp2^+/− Jae^, and their comparison were never achieved before. The simplest hypothesis is that this is due to the complete absence of Mecp2 in Mecp2^y/−^ males and by the presence of a mosaic-like expression of Mecp2^+/− Jae^ in females. Along the same line, a previous work that has attempted to compare the morphological organization of dendrites and dendritic spines in Mecp2^y/−^ male and Mecp2^+/−^ female showed clear differences in several neuronal parameters between the two genotypes [63].

Importantly, this study reveals that both mouse strains present synaptic alterations leading to a common over-activation of JNK. For instance, for the first time, we characterize Shank3 in Mecp2 mutant mice. Shank3 encodes an abundant postsynaptic scaffold protein, highly enriched in the glutamatergic synapses in the human and murine central nervous system (CNS), important in regulating synaptic structure and function. In both Mecp2^y/−^ and Mecp2^+/− Jae^ models, we here report Shank3 level deregulation.

This study shows for the first time that JNK kinase in the brain is activated both in the absence (Mecp2^y/−^) and in the female mosaicism expression of Mecp2 mutation and wild type allele (Mecp2^+/− Jae^ mice) and the specific inhibition of JNK reverses RTT signs/symptoms in both mouse models.

We would like to underline that the excitatory postsynaptic compartment anomalies have been described in several neurological disorders associated with cognitive decline, including typical aging, Alzheimer’s and Huntington diseases, schizophrenia, neurodevelopmental intellectual disabilities, autism spectrum disorders, and in RTT as well [64–71]. Synaptic dysfunction is the first degenerative event in brain diseases and represents an important therapeutical window of intervention. In fact, potential treatment able to promote the maintenance of biochemical markers of the PSD region and to restore the activity of spines will offer one of the most exciting therapeutic agents against many different brain diseases. Since JNK-specific inhibition prevents RTT synaptopathy and rescues behavioral and breathing defects, treatments that target the JNK pathway represent strong candidates against RTT and other neurodevelopmental disorders.

It will be important, in future studies, to assess the molecular link between Mecp2 and JNK. This could provide important information on how the Mecp2 loss/mutation induces JNK activation. The connection between Mecp2 and JNK pathway is a very intriguing issue; some indications from the literature showed that JNK inhibition, with SP600125 (chemical inhibitor), decreased the bindings of MeCP2 and histone-3 trimethyl K9 to the MOR promoter indicating a transcriptional regulation of MOR by JNK inhibition [72]. In addition, our unpublished results of the system biology analysis on MECP2 network identify JNK as a signaling pathway implicated in RETT synaptic dysfunction (Borsello unpublished). Further investigations to identify the relation between Mecp2 and stress JNK pathway are currently ongoing.

Importantly, the results on neurons derived from iPSCs (hiPSCs) are the first proof of the principle of JNK’s role in the Human RTT. The hiPSC technology is important to discover druggable targets in human diseases and for testing the efficacy and specificity of new therapeutic compounds. We find JNK activation in hMECP2mut-differentiated neurons, and this correlates with an increased neuronal death, importantly, the isogenic hiPSCs show control levels of JNK and no cell death. The D-JNKI1 treatment of hMECP2mut neurons inhibits JNK activation and prevents neuronal death, indicating its clinical relevance. The neurons differentiated from hMECP2mut can be used as a simple model to test drugs against different MECP2 mutations. In this initial study, the characterization of neuronal cells is not complete, since it is focused only on JNK signaling. However, a more complete research to better analyze the neuronal populations, including synapse and dendritic spine density, size, and number in hMECP2^mut^ compared to the control hMECP2, is programmed. The hMECP2-iPSCs represent an opportunity to explore disease mechanisms in a simple model that allows to study different point mutations of the MECP2 gene, verifying the potential differences with the animal models. In addition, they may represent intriguing tools for personalize medicine being also used in drug screening.

The complexity of the RTT clinical phenotypes and the different mechanisms involved can be very discouraging to cure this neurological disorder; however, taking advantage of hiPSCs is a good and relatively simple strategy to enlighten cellular pathways and biochemical interactions in human disease.

Currently, there is no cure for RTT, and medical management is aimed to provide symptomatic relief for patients through a multidisciplinary approach. Some of the medical concerns that need to be addressed in RTT patients include a few disorders, including breathing irregularities such as apnea, hyperventilation, and breath-holding [12]. Successful management of these irregularities can be difficult, and sudden death in patients with RTT as a result of an autonomic dysfunction is a potential risk factor. However, precautions should be taken to avoid medications that alter breathing patterns, for example, opioid medications [73]. Management of these conditions can substantially improve the quality of life of RTT patients and should not be overlooked [74]. In such as context, these findings are encouraging for further studies in animal models that overt breathing abnormalities focused on the efficacy of preventive administration of the D-JNKI1 inhibitor reducing breathing disorders and potentially encouraging to undertake clinical studies on brainstem modulation of breathing in patients with RTT.

Finally, we suggest that these results can open the real possibility to D-JNKI1 clinical application. Indeed D-JNKI1 (commercially available as XG102/AM11) is already in phase III clinical trials aimed at assessing the ability of the substance to inhibit intraocular inflammation and reduce pain in patients undergoing cataract surgery, as well as on the treatment of hearing loss [75]. Although there is no evidence of side effects of the D-JNKI1 inhibitor in mice [26, 28, 33, 62], the passage to humans could lead to unwanted effects, in fact, chronic clinical studies, so far, are restricted to topical usage [76]. One of the main limitation/problems of D-JNKI1/XG102/AM111 is the specificity of the delivery: the peptide is not cell- or tissue-specific that may imply different potential side-effects. A chronic and systemic inhibition of the JNK pathway could generate side effects since the JNK family regulate a myriad of cellular functions, some not linked to the pathophysiology of diseases. We think that a possible solution can be to target just JNK3. The kinase is the brain-specific isoform of JNKs and the most responsive to stress stimulations [77]. While JNK1 and JNK2 are ubiquitously expressed throughout the body and play roles in cell proliferation and other important physiological functions [78]. The specific inhibition of the JNK3 isoform offers the advantage of (i) acting only in the nervous tissue (not in the rest of the body) and (ii) quivering only the pathological action, mediated by the stress stimuli (JNK3 is the most responsive JNKs to stress-stimuli), thus preserving the physiological actions of the JNK family and avoiding potential side-effects.

## Conclusion

The results of our investigations, on the two Rett mice models, confirmed the dysregulation of the excitatory post-synaptic marker levels in the PSD region and the JNK activation in the post-synaptic protein-enriched fraction (TIF fraction) in different brain areas. The specific JNK inhibitor, D-JNKI1, is able to recover the synaptic dysfunction on both models demonstrating the JNK key role in both Mecp2-knockout male and Mecp2-heterozygous female mice.

Translational value is given then by the hiPSC results, proving that only the hiPSCs mut neurons showed a larger increase of P-c-jun levels associated with cell death, which is not observed in the isogenic control allele hiPSCs wt. D-JNKI1 in hiPSCs mut neurons inhibits neuronal death induced by Mecp2 mutation.

These results strongly suggest that JNK plays a key role in RTT and its specific inhibition tackle RTT in the two mouse models and in a human model as well. For all these reasons, we conclude that JNK is a potential therapeutic target in Rett syndrome.

## Methods

### Animal procedures

We used two mouse models of RTT: Mecp2^tm1.1 Bird^ mice and Mecp2^tml.l Jae^ mice (Jackson Laboratory, Bar Harbor, Maine). Knockout Mecp2^tm1.1Bird^ male mice were on a C57BL/6 background and age-compatible wild-type male mice (C57BL/6) served as controls. Heterozygous female Mecp2^tml.lJae^ mice with exon 3 deletion in Mecp2 were crossed to C57BL6 for one generation, followed by breeding among offspring and then maintained on a mixed background; we used age-matched wild-type females as controls in all experimental conditions. Genotyping was done by PCR using a protocol provided by Jackson Laboratory and GoTaq®G2 Flexi DNA polymerase kit (Promega, Madison, USA). Mice were bred at IRCCS Mario Negri Institute for Pharmacological Research in a specific pathogen-free (SPF) facility with a regular 12:12 h light/dark cycle (lights on 07:00 a.m.), at a constant room temperature of 22 ± 2°C, and relative humidity 55 ± 10%. Animals were housed in standard mouse cages with water and food ad libitum. Procedures involving animals and their care were in accordance with national and international laws and policies. The Mario Negri Institute for Pharmacological Research (IRCCS, Milan, Italy) Animal Care and Use Committee (IACUC) approved the experiment, which was conducted according to the institutional guidelines, in compliance with Italian laws. The scientific project was approved by the Italian Ministry of Health (Permit Number 43/03).

The experimental scheme was composed of 8 groups: untreated wild-type and Mecp2^y/−^ male mice, D-JNKI1-treated wild-type and Mecp2^y/−^ male mice, untreated wild-type and Mecp2^+/− Jae^ female mice, and D-JNKI1-treated wild-type and Mecp2^+/− Jae^ female mice. For behavioral tests, we used *n*=10 wild-type male mice, *n*=10 Mecp2^y/−^ male mice, *n*=8 wild-type female mice, and *n*=8 Mecp2^+/− Jae^ female mice; for biochemical analysis (TIF and Western blots), we analyzed *n*=10 animals of Bird strain and *n*=10 animals of Jaenisch strain for each experimental group.

### Animal welfare

Animals (*n*=25 wild-type male mice, *n*=25 Mecp2^y/−^ male mice) were monitored daily for well-being and welfare-related disease symptoms. Bodyweight was recorded weekly as well as food and water intake. Bodyweight loss was calculated as the difference (grams) from the maximum weight recorded for each animal.

### Animal treatment

Based on previous works described in the D-JNKI1, dose and paradigm of administration were decided. Animals were randomized assigning random numbers to experimental groups and also with respect to disease severity. In brief, all mice (Mecp2^y/−^ or wt) were injected intraperitoneally with D-JNKI1 (22 mg/kg) every 28 days from 3 to 7 weeks of age [62]. Their weight was recorded before each treatment. Mice were always treated at the same time of day (9:00–10:00 A.M.) in a randomized order in a specific room inside the animal facility. Every single mouse was our experimental unit. Heterozygous Mecp2^+/− Jae^ female and wt littermates were treated with D-JNKI1 (22 mg/kg, i.p.) at 16 and 20 weeks of age. After behavioral trials were completed, animals were euthanized and the brains were dissected for biochemical analysis.

### Behavioral tests

All the behavioral tests started 24h after the D-JNKI1 injection.

#### Rotarod test

Treated and untreated mice were tested weekly for any deficits in Rotarod performance by the same operator who was blind to the treatment given. Analyses started from 3 to 7 weeks of age for Mecp2^y/−^ and wt mice. Rotarod testing was done using the accelerating Rotarod apparatus (Ugo Basile 7650 model, Ugo Basile, Varese, Italy) as previously described. Once the animals were positioned on the rotating bar, time was started and the rod was accelerated at a constant rate of 0.3 rpm/s from 3 to 30 rpm for a maximum of 5 min. The time (seconds) at which the animal fell from the bar was recorded. Three trials were run for each animal, with a 5-min rest between trials, and the longest retention time was recorded. The mean latency to fall during the session was calculated and used in subsequent analysis.

#### Open field and spontaneous locomotor activity

The open field (OF) test is used to examine general locomotion, as well as exploration, and the relative level of anxiety by exposing mice to a novel and open space [79–81]. We used a gray Perspex OF box (40 × 40 × 40 cm) with the floor divided into 25 (8 × 8 cm) squares. After allowing the mice to acclimatize to the testing room for 30 min, they were placed in the behavioral box in order to reduce their reactions to the novel environment. They were placed in the center of the floor, defined as a “starting point,” and their behavior was tracked with the activity monitoring system Ethovision (Noldus, Wageningen, Netherlands) for 5 min. This short time was chosen to avoid further stress to mutant mice (*n*=10/experimental group). The parameters analyzed as measures of spontaneous locomotor activity, exploratory activity, and state of anxiety were the duration of movements divided into the number of internal (the nine central squares) and external (the sixteen peripheral squares) square crossed, the time spent in the central and outer areas of the open field, the overall distance traveled by the mice, the number and duration of rearings (standing on the hind legs with the front limbs either against the wall or free in the air [81], the number and duration of self-grooming [82] (rubbing the body with the paws or mouth and rubbing the head with paws).

These protocols were used to test the Mecp2^y/−^ and Mecp2^+/− Jae^ and the relative control mice. In heterozygous Mecp2^+/− Jae^ females, locomotor activity was recorded for 30 min. The behavioral parameters recorded were the overall distance traveled and the distance traveled in the periphery and in the center of the open-field arena.

### Whole-body plethysmography (WBP) analysis

Unrestrained Mecp2^y/−^ and wt mice were placed in a WBP recording chamber (Emka Technology, Paris). After a habituation period of 15 min, a baseline recording was established for 30 min. Mice were then removed from the chamber, injected intraperitoneally with the D-JNKI1 peptide (22 mg/kg). Animals were recorded 24 h after the injection and then once a week from 6 to 9 weeks of age. Analysis was performed using IOX2 software (EMKA Technologies, Paris). Apnea was considered only if the end-expiratory pause was ≥ 0.8 s. Only points of motion-free recording were analyzed. The periods of the movement were removed automatically by the apnea software.

### Biochemical analysis: subcellular fractionation (TIF)

For biochemical analysis, at the end of behavioral tests, mice were euthanized by cervical dislocation [83, 84]; the brains were removed and specific brain areas, cortex, hippocampus, and cerebellum, were dissected and stored at –80°C until sample processing. Subcellular fractionation was as reported in the literature, with minor modifications, for the cortex, hippocampus, and cerebellum from Mecp2^y/−^, Mecp2^+/− Jae^, and control mice. Briefly, the tissue was homogenized with a glass-glass Potter apparatus in 0.32 M ice-cold sucrose (S0389, Sigma-Aldrich, Darmstadt, Germany) buffer containing the following concentrations (in mM): 1 HEPES (H3375, Sigma-Aldrich, Darmstadt, Germany), 1 MgCl2 (M8266, Sigma-Aldrich Darmstadt, Germany), 1 EDTA (324503, Sigma-Aldrich, Darmstadt, Germany), 1 NaHCO_3_ (S5761 Sigma-Aldrich, Darmstadt, Germany), and 0.1 PMSF (P7626, Sigma-Aldrich, Darmstadt, Germany) at pH 7.4, with a complete set of protease inhibitors (4693124001, 04906837001, Roche Diagnostics, Basel, Switzerland) and phosphatase inhibitors (4693124001, 04906837001, Roche Diagnostics, Basel, Switzerland). Samples were centrifuged at 1000×*g* for 10 min. The supernatant (S1) was then centrifuged at 3000×*g* for 15 min to obtain a crude membrane fraction (P2 fraction). The pellet was dissolved in a buffer containing 75 mM KCl (P5405, Sigma-Aldrich, Darmstadt, Germany) and 1% Triton X-100 (X100, Sigma-Aldrich, Darmstadt, Germany) plus protease and phosphatase inhibitors (4693124001, 04906837001, Roche Diagnostics, Basel, Switzerland) and centrifuged at 100,000×*g* for 1 h. The supernatant was stored and referred to as TSF (S4). The final pellet (P4), referred to as TIF, was homogenized in a glass Potter apparatus in 20 mM HEPES (H3375, Sigma-Aldrich, Darmstadt, Germany) with a complete set of protease and phosphatase inhibitors (4693124001, 04906837001 Roche Diagnostics, Basel, Switzerland) and stored at −80°C until processing [85].

### Western blot

Protein concentrations were quantified using the Bradford Assay (5000006, Bio-Rad Protein Assay Hercules, California, USA): 10 μg of total homogenate and 5 μg of TIF extracted proteins were separated by 10% SDS polyacrylamide gel electrophoresis. PVDF membranes (1620177, Bio-Rad, Hercules, California, USA) were blocked in Tris-buffered saline 5% no-fat milk powder (70166, Sigma-Aldrich, Darmstadt, Germany) and 0.1% Tween 20 (P1379, Sigma-Aldrich, Darmstadt, Germany) (1 h, RT). Primary antibodies were diluted in the same buffer (incubation overnight, 4°C) using anti-P-JNKs (1:1000, BK9251S, Cell Signaling, Danvers, MA, USA), anti-JNKs (1:1000 BK9252S, Cell Signaling, Danvers, MA, USA), anti-p-c-Jun (1:1000, 06-659, Millipore, Bedford, MA, USA), anti-c-Jun (1:1000, BK9165S, Cell Signaling, Danvers, MA, USA), anti-NMDA Receptor 2A GluN2A (1:2000, BK4205S, Cell Signaling, Danvers, MA, USA), anti-NMDA Receptor 2B GluN2B (1:2000, BK14544S, Cell Signaling, Danvers, MA, USA), anti-Glutamate Receptor 1 (AMPA subtype) GluA1 (1:1000, BK13185S Millipore, Bedford, MA, USA), anti-Glutamate Receptor 2 (AMPA subtype) GluA2 (1:1000, MAB397 Millipore, Bedford, MA, USA), anti-postsynaptic density protein 95 (1:2000, CAY-10011435-100, Cayman Chemical Company, Ann Arbor, Michigan, USA), anti-PSD93 (1:1000, AB2930, Abcam, Cambridge, UK), anti-Drebrin (1:1000, BSR-M05530, Boster, Pleasanton, CA, USA), anti-Shank3 (1:1000, 64555, Cell Signaling, Danvers, MA, USA), and anti-Actin (1:5000, MAB1501 Millipore, Bedford, MA, USA). Blots were developed using horseradish peroxidase-conjugated secondary antibodies (SC-2357, SC-516102, Santa Cruz Biotechnology, CA, USA) and the ECL chemiluminescence system (w1001, Promega, Madison, USA). Western blots were quantified by densitometry using Quantity One software (Bio-Rad, Hercules, California, USA).

### Immunohistochemistry

Mice were deeply anesthetized using ketamine (75 mg/kg) and medetomidine (0.5 mg/kg) then perfused via the ascending aorta. The brains were removed from the skull and post-fixed for 90 min at 4°C, then transferred to 20% sucrose in phosphate-buffered saline (PBS) for 24 h at 4°C, frozen in n-pentane for 3 min at −50°C and stored at −80°C until assay. Serial sagittal sections (30 μm) were cut on a cryostat and then collected in 1X PBS for immunohistochemistry. Brain slices were incubated for 1 h at room temperature with blocking solutions (0.4% Triton X-100 plus 10% NGS) and then overnight at 4°C with the primary antibody anti-p-c-Jun (1:250, 06-659, Millipore, Bedford, MA, USA). After the incubation with the primary antibody, slices were incubated for 1 h at RT with a biotinylated secondary antibody (1:200, Vector Labs, Burlingame, CA, USA) and then in avidin-biotin-peroxidase complex (Vector Laboratories) and diaminobenzidine (Sigma-Aldrich, Italy).

### LDH assay

The cell medium was collected and the release of LDH in the medium was quantified to assess cell viability, using the cytotoxic 96 non-radioactive cytotoxicity assay kit (Promega, Madison, USA, USA).

### iPSCs and iPSC-derived neurons

We analyzed three iPSC clones derived from fibroblasts of a female patient with Thr158Met mutation in the MECP2 gene: two clones expressing the mutated MECP2 allele (2271#22 and 2271#1) and one expressing the normal allele (2271#2) due to X-chromosome inactivation, which was used as a partial isogenic control [14]. An iPSC line from a second MECP2-mutated patient with a p.Arg306 Cys mutation was obtained from James Ellis (University of Toronto) [86]. As additional controls, we used two iPSC clones from a healthy newborn male and one healthy female child. All iPSC lines were derived using the Yamanaka’s classic retroviral approach [87] and characterized according to standard criteria [88]. Neurons were differentiated from mutated and control iPSCs lines as previously reported [14]. On day 30 of terminal differentiation, neurons for quantitative analyses were isolated by immuno-magnetic sorting using anti-CD24 antibodies (130-095-951, Miltenyi Biotec, Bergisch Gladbach, Germany) [89].

### Drug treatment

Based on previous in vitro experiments described [26, 27], control and mutated neurons were treated with D-JNKI1 at 2 μM concentration starting on day 28 of terminal differentiation. On day 30, neurons were isolated as described above to obtain proteins for Western blot analysis. Proteins were extracted with sucrose 1.09 g (S0389, Sigma-Aldrich, Darmstadt, Germany), NaHCO_3_ 1 mM (S5761, Sigma-Aldrich Darmstadt, Germany), MgCl2 1mM (M8266, Sigma-Aldrich Darmstadt, Germany), Hepes 1 mM (H3375, Sigma-Aldrich, Darmstadt, Germany), NaF 10 mM (201154, Sigma-Aldrich, Darmstadt, Germany) Triton X-100 0.1% (X100, Sigma-Aldrich, Darmstadt, Germany), DTT 1mM (GE17-1318-01, Sigma-Aldrich, Darmstadt, Germany), NaOH 1 mM (S8045, Sigma-Aldrich, Darmstadt, Germany), PMSF 1 mM (P7626, Sigma-Aldrich, Darmstadt, Germany), and protease inhibitor (4693124001, 04906837001, Complete; Roche Diagnostics, Basel, Switzerland).

### Statistical analysis

Statistical analysis was done with the Graph Pad Prism 6 program. Data were expressed as mean ± SEM with statistical significance *p*< 0.05. For comparison between multiple groups (body weights, behavioral, and breathing parameters), two-way ANOVA for repetitive measurements followed by Bonferroni post hoc test was used; for comparison between two groups (biochemical analysis), two-way ANOVA followed by Bonferroni post hoc test was used; for comparison between two groups (genotype or treatment), Student’s *t* test followed by Tukey’s post hoc test was used.

## Supplementary Information

**Additional file 1.** Fig. S1 Brainstem of Mecp2^y/-^ male mice presents a strong p-c-jun immunoreactivity compared to wild-type mice. Representative section of p-c-jun immuno-staining in the brain of wt (a) and higher magnification (b) of a brainstem area show a low number of p-c-jun immunopositive cells. Representative section of p-c-jun immuno-staining in the brain of Mecp2^y/-^ (c) and higher magnification (d) of a brainstem area show an higher number of p-c-jun immunopositive cells

**Additional file 2.** Images of western blots

**Additional file 3.** Data relative to Figure 5

## Data Availability

The datasets used and/or analyzed during the current study are available from the corresponding author on reasonable request in https://zenodo.org/record/5608558#.YXrZnsZabJ8 (see references 90).

## Abbreviations

RTT: Rett syndrome
JNK: c-Jun N-terminal kinase
hiPSCs: Human-induced pluripotent stem cells
CNS: Central nervous system
Mecp2^y/−^: Mecp2-knockout male mice
Mecp2^+/−^ Jae: Mecp2-heterozygous female mice
MECP2mut: MECP2-mutated iPSCs
MECP2wt: MECP2-wild-type allele iPSCs
f: Frequency
Ti: Time of inspiration
Te: Time of expiration
TIF: Triton-insoluble fraction
GluN2A: *N*-methyl-d-aspartate receptor 2A
GluN2B: *N*-methyl-d-aspartate receptor 2B
MAP1B: MT-associated protein 1B
SPF: Specific pathogen-free
IACUC: Animal Care and Use Committee
OF: Open field test
