# Supplementary figures and images for: JNK signaling provides a novel therapeutic target for Rett syndrome
Source: BMC Biol. 2021 Dec 16;19:256. doi: 10.1186/s12915-021-01190-2 (PMC8675514; doi:10.1186/s12915-021-01190-2)

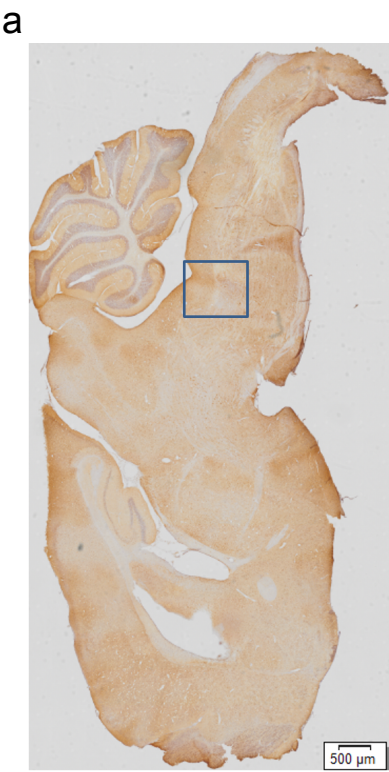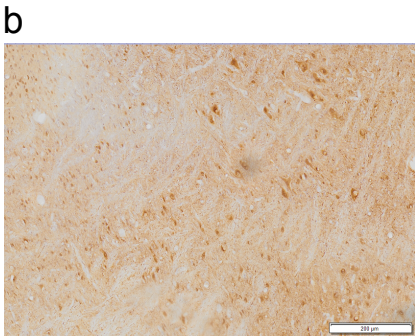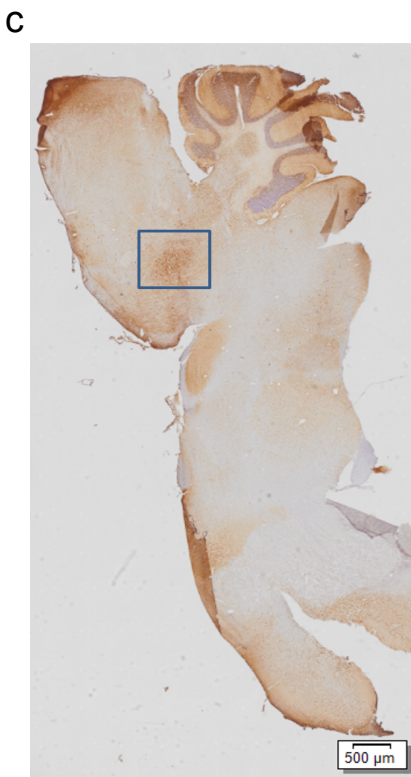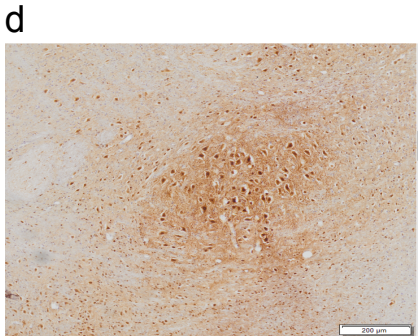

SUPPLEMENTARY FIGURE 1

Supplement: Supplementary file 1 — Additional file 1. Fig. S1 Brainstem of Mecp2y/- male mice presents a strong p-c-jun immunoreactivity compared to wild-type mice. Representative section of p-c-jun immuno-staining in the brain of wt (a) and higher magnification (b) of a brainstem area show a low number of p-c-jun immunopositive cells. Representative section of p-c-jun immuno-staining in the brain of Mecp2y/- (c) and higher magnification (d) of a brainstem area show an higher number of p-c-jun immunopositive cells [file 12915_2021_1190_MOESM1_ESM.pdf]
