# Supplementary material for: JNK signaling provides a novel therapeutic target for Rett syndrome
Source: BMC Biol. 2021 Dec 16;19:256. doi: 10.1186/s12915-021-01190-2 (PMC8675514; doi:10.1186/s12915-021-01190-2)
Supplement: Supplementary file 2 — Additional file 2. Images of western blots [file 12915_2021_1190_MOESM2_ESM.pdf]

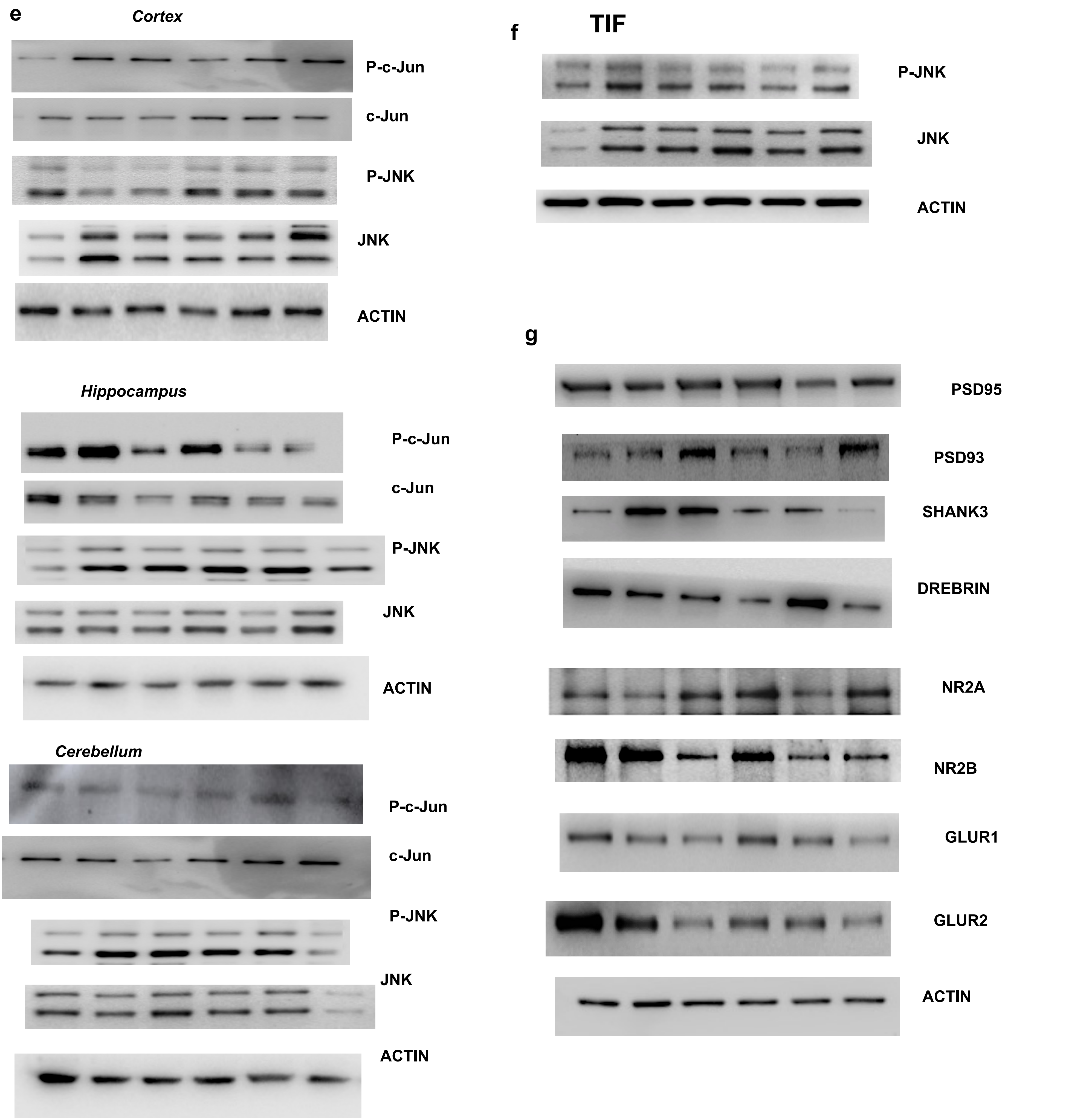

All membranes were cut in order to incubate at the same time different antibodies

FIGURE 1

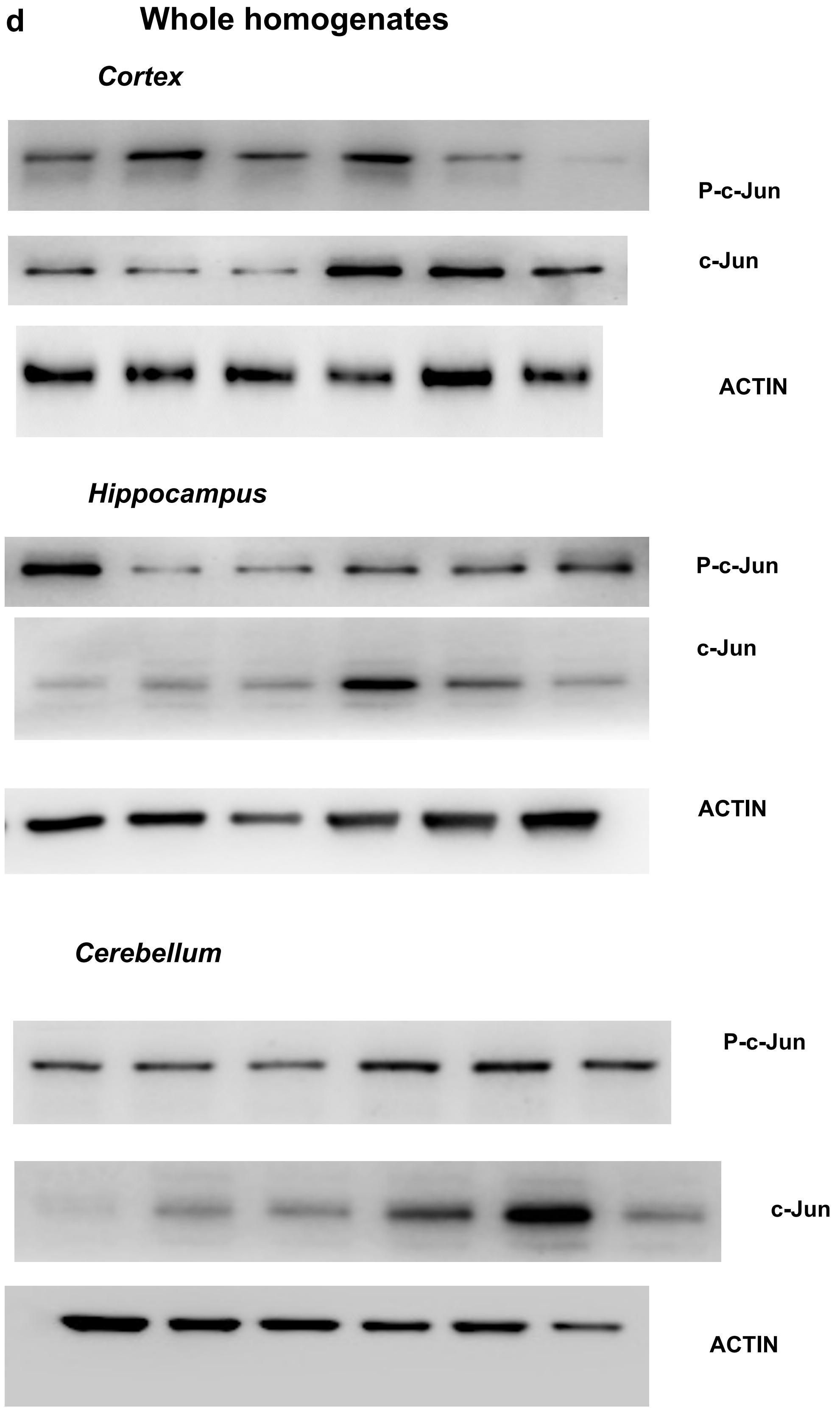

FIGURE 2

All membranes were cut in order to incubate at the same time different antibodies

b **TIF**

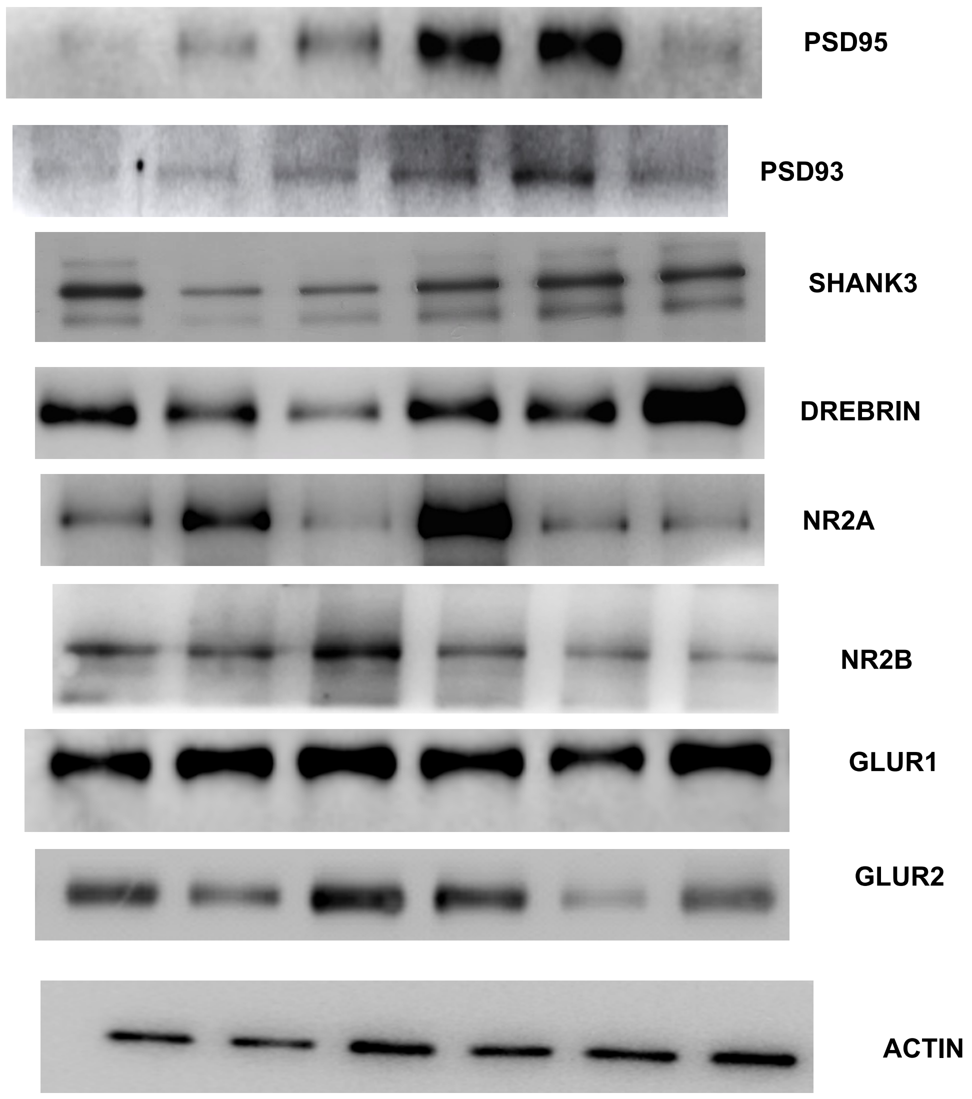

a

FIGURE 3

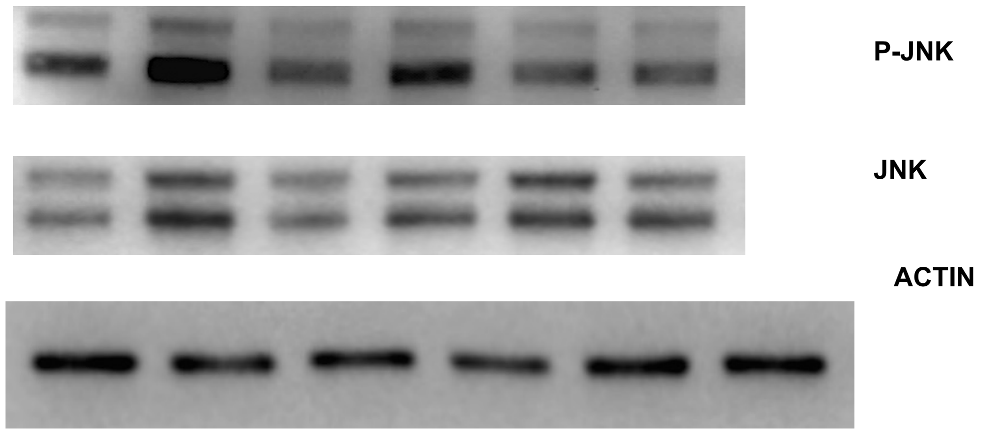

All membranes were cut in order to incubate at the same time different antibodies

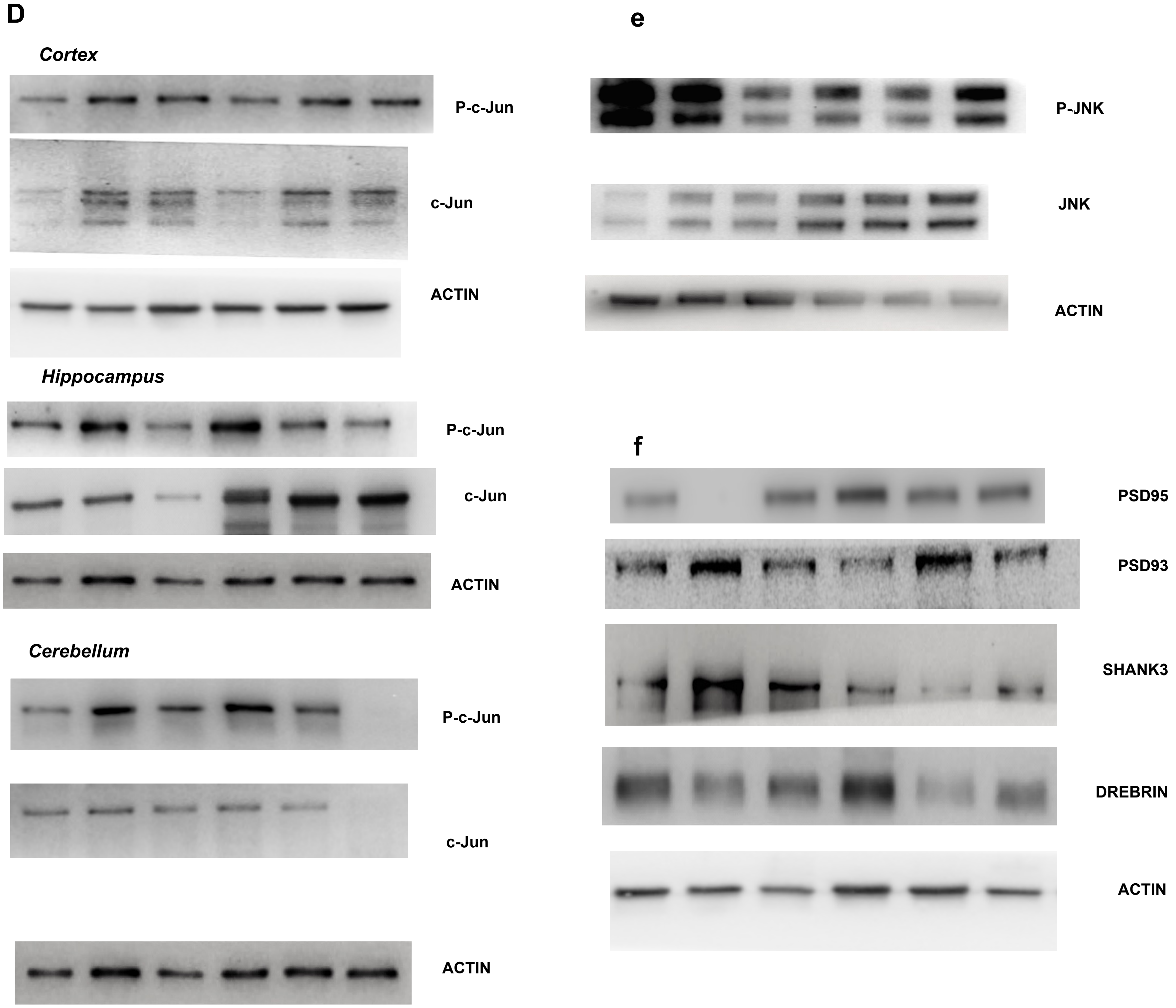

All membranes were cut in order to incubate at the same time different antibodies

FIGURE 4

b

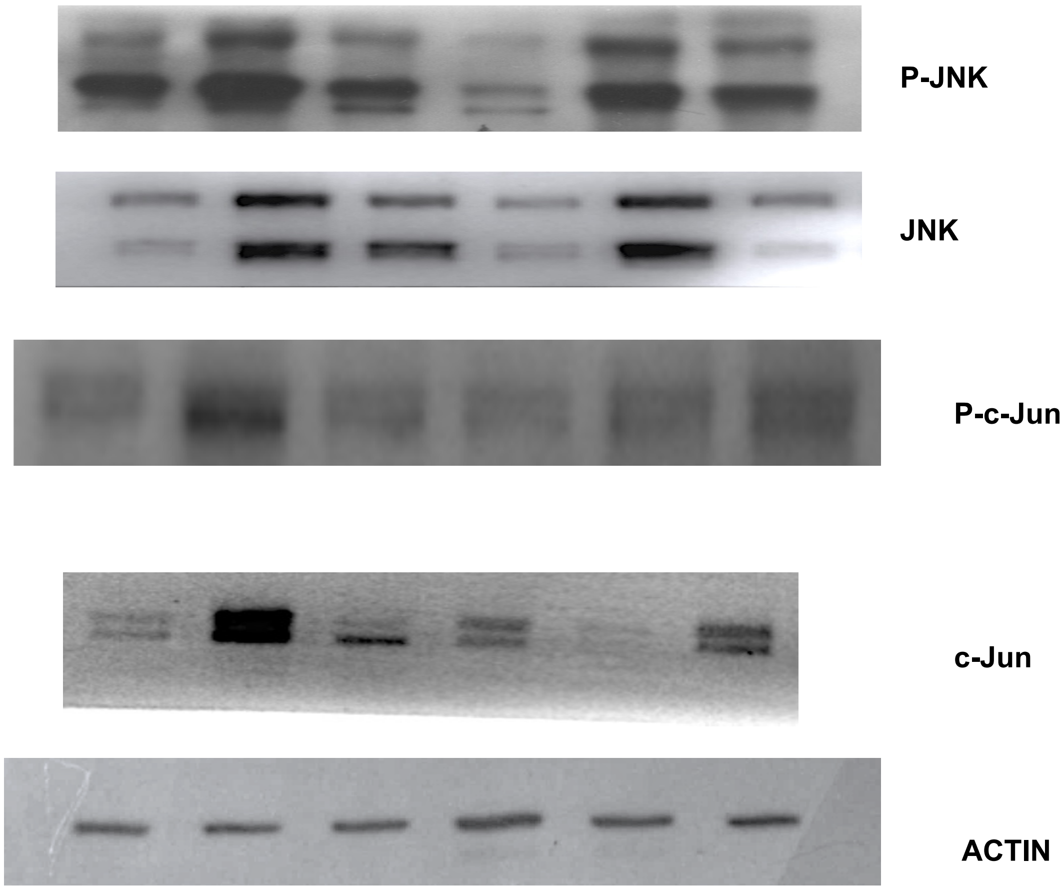

All membranes were cut in order to incubate at the same time different antibodies

FIGURE 5
